# Supplementary material for: Married women’s autonomy in modern contraceptive utilization in Kutaber district, Northeast Ethiopia
Source: PLOS Glob Public Health. 2025 May 23;5(5):e0004671. doi: 10.1371/journal.pgph.0004671 (PMC12101775; doi:10.1371/journal.pgph.0004671)
Supplement: Attachment — Submitted filename: Ethiopian Womens Autonomy in Contraceptive Use_Review.docx [file pgph.0004671.s001.docx]

This study considered autonomy in use of modern contraceptives among married Ethiopian women in a Kutaber district of Ethiopia. Authors have done a great job with the study.

Authors should consider placing findings from this study in context of findings not only from Ethiopia but also other countries in sub-Saharan Africa and countries like Ethiopia, globally. Authors need to provide strong justification for why this study is necessary and the particular gaps that this study is filling.

Introduction

- Provide references right after the sentences in lines 49, 51, and 54. Generally, it is best practice to provide in-text citation immediately after factual statements, even if you have to repeat the same citation in the next line.
- Line 85, do you mean women’s lack of freedom in decision-making? Specify why they are not engaging in decision making by qualifying with an adjective.
- Line 94, “according to the United Nations..”
- Line 98, provide citations for these Ethiopian studies
- Authors mentioned that “most Ethiopian studies..”. Highlight the Ethiopian studies that have looked at the variables: women’s freedom of movement and financial control and what the gaps are that your study is filling.

Materials and Methods

- Household decision making power: were the questions pre-determined from the literature or were they created by researchers? If created, what was the rationale for these 6 questions? Were they pilot-tested?
- Same questions as above apply to the “freedom of movement”, “control over finance”, constructs.
- For the construct on “knowledge of modern contraceptive methods”: specify is this was binary (i.e., yes or no) or continuous and if so, what was the scale
- Line 200: women who **listened to the radio**
- For media exposure: did you consider social media? If not, consider this as a limitation in your discussion section
- Include in this section, how the variables on reproductive history and modern contraceptive use history, were measured

Discussion

- Line 318, further explain how these discrepancies can create such differences in the results…are these parts more religious, cultural etc?
- Same for line 321-322.
- Are there other studies that can be cited to show that unmarried women have more contraceptive use autonomy?
- Why are you seeing higher numbers than reported by the EDHS for categories of women using contraceptives on the basis of their husbands’ decisions alone and those that stopped using because of their husbands’ decision alone? Provide more critical insights.
- Provide references for line 343
- Empowering women by doing what…line 345
- Provide references for line 353-358
- Provide references for lines 361- 364 “this might be because when a couple lives together for a longer period…”
- Line 365 is awkward…”live children”? or children..is there a reason why you are distinguishing between having children and not having children by specifying they are living?
- Lines 367 to 369 does not provide a lot of insight..edit and ensure it is helping us understand why more children can mean more autonomy for the mother as opposed to “spouse”. More so, is there evidence to support this line of thought?
- Authors should include a section in the manuscript’s discussion titled “implications”. What are the implications of this study for global public health, policy making, community health?

Limitations

- More so, did your limitations consider that women may be using other non-modern, and perhaps traditional methods of contraceptives that may not be measured by this study?
- Why is it important for women to use modern contraceptives over more indigenous ones? Western, modern versus traditional..what is the rationale here? Comment on this in the limitations

Conclusion

- When you say women’s autonomy was low..in comparison to..? What is the baseline for measurement here?
- Future research may also consider how attitudes towards these contraceptives influence utilization and autonomy
