# Supplementary material for: Married women’s autonomy in modern contraceptive utilization in Kutaber district, Northeast Ethiopia
Source: PLOS Glob Public Health. 2025 May 23;5(5):e0004671. doi: 10.1371/journal.pgph.0004671 (PMC12101775; doi:10.1371/journal.pgph.0004671)
Supplement: Attachment — Submitted filename: Response to Reviewers.docx [file pgph.0004671.s003.docx]

**Date: January 05, 2025**

**Rebuttal letter**

PGPH-D-24-02291

Married women’s autonomy in modern contraceptive utilization in Kutaber district, Northeast Ethiopia

PLOS Global Public Health

Dear Editor:

We would like to express our appreciation for your extensive support for the progress our manuscript. Almost all of the comments and suggestions were acceptable, and the necessary changes were made to each point. Your feedback and suggestions help us for enhancement our manuscript and allow us to go forward. So, once again, thank you for your exhaustive review of the manuscript. Our point-by-point replies to the comments and questions are detailed, and some of these improvements are highlighted. Furthermore, the specifics of modifications are displayed by tracking changes in the related file to the submission system and highlighting in the revised manuscript. We eagerly await your response in order for the manuscript to move smoothly in the next future.

“Best regards”

Getachew Amanu

| **S.No** | **Comments/ Recommendations** | **Response** |
| --- | --- | --- |
| 01 | Placing your findings within a broader context by comparing them with similar studies from Sub-Saharan Africa and other global contexts. | Thank you for your concern and great suggestions.  We made comparisons with similar studies conducted in Sub-Saharan Africa countries. |
| 02 | Clearer definitions and consistent usage of terms like autonomy, decision-making power, and empowerment. | Thank you! We have clearly define and operationalized the term autonomy and decision-making power and used them consistently. |
| 03 | In terms of methodology, more detail is needed regarding the development and validation of the measures for decision-making power, freedom of movement, and financial control | The questions for the development of decision-making power, freedom of movement, and financial control variable were constructed from items in the EDHS 2016. |
| 04 | The process of data collection from illiterate participants and how privacy was maintained should be clarified. | Even if 52.7% of respondents were illiterate, the data collectors collected the data through face-to-face interviews using a structured questionnaire. |
| 05 | Discussing checks for multicollinearity and model fit in your regression analysis would further enhance the validity of your results. | Thank you! We have checked for multicollinearity and model fitness. VIF <4.0 and Hosmer-lemeshow goodness of test (Chi-square:8.799, P-value: 0.36). |
| 06 | In the discussion, a more nuanced interpretation of the discrepancies in contraceptive autonomy compared to EDHS data is needed, with an emphasis on potential cultural, religious, or regional factors. | Thank you! We made nuanced interpretation for the discrepancies between our study and the EDHS 2016. |
| 07 | I also suggest adding a section on the broader implications of your study for global public health and policy. | Thank you for your great concern. We have added the implications of the study in the discussion section. |
| 08 | A careful review of the manuscript’s English language and grammar is recommended. | We carefully reviewed the manuscript for English language and grammar errors, correcting all issues to improve flow and readability. |
| 09 | Finally, the limitations section should address the potential underreporting of non-modern contraceptive use and clarify the rationale for focusing on modern methods. | While traditional contraceptive methods play a role in family planning, their limitations in effectiveness and reliability warrant a focus on modern alternatives. |
| 10 | In the conclusion, specify the baseline against which autonomy is measured to provide clearer context. | We concluded that the prevalence of autonomy in the study area was higher compared to the recent national study in Ethiopia using the EDHS 2016. |

**Response to Editor’s Comment**

| **S.No** | **Comments** | **Response** |
| --- | --- | --- |
| 01 | The authors stated that all modern family planning services are provided to the clients for free at public health facilities (lines 109-110); if so, studying many variables related to women's autonomy on money or income might not be relevant because they do not go with the aims of the study. | Thank you for your comment.  Even if modern family planning methods are provided for free in public health facilities, private health facilities charge for these services. Additionally, women still need money for transportation expenses to reach these facilities. |
| 02 | It is better to use standard scoring tools rather than citing previous studies to measure some parameters such as control over finance and knowledge of modern contraceptive methods (Lines 184-195). | Great!  We used the scoring tools of the 2019 Mini Ethiopia Demographic and Health Survey (EDHS) to measure control over finances and knowledge of modern contraceptives. |
| 03 | In the data collection tool, it is stated that the authors used questionnaires as a tool. However, in the result part, 52% of the participants did not have formal education (could not read and write). How were their data obtained? please justify it. | Even if 52.7% of respondents were illiterate, the data collectors collected the data through face-to-face interviews using a structured questionnaire. |
| 04 | Regarding privacy, it is better to justify the mechanisms used to maintain the privacy of the study participants during data collection. | Thank you for your concern.  The interviews were conducted in an isolated area away from household members to maintain the respondents' privacy. |
| 05 | What scientific standards were used to make categories of the number of children the study participants had (1-2, 3-4 or >5, etc)? please justify. | We categorized the number of children in the same way as it was categorized in the 2016 EDHS for the purpose of comparison. |

**Response to Reviewer #1 comments**

**Reviewer #2 Comments**

| **S.No** | **Comments** | **Response** |
| --- | --- | --- |
| 01 | Define autonomy clearly as it relates to family planning, and use consistent language to discuss this concept throughout the introduction to avoid confusion. | Thank you for your comment.  We revised the definition of autonomy in contraceptive use and used the term consistently. |
| 02 | Lines 53-59 provide overlapping statistics on maternal mortality, neonatal mortality, and fertility rates. Summarizing or consolidating some of this information can help keep the reader focused on the core issues. | Great!  We summarized in the revised manuscript. |
| 03 | Some phrases are repeated or redundant. For example, lines 77-80 describe the benefits of autonomy in similar ways to prior sentences. Condensing these could improve flow and readability. | Thank you!  We condensed the redundant phrases. |
| 04 | Selecting six kebeles from 23 using random sampling ensures representation across different sub-districts, but it is essential to specify if each kebele has a similar or varying demographic profile. | Thank you for your concern.  The socio-demographic characteristics of the 23 kebeles (sub-districts) do not vary significantly. |
| 05 | Allocating the sample size proportionally to each kebele is appropriate and helps maintain representativeness. However, it is unclear how households with eligible women were listed or verified. | The households with eligible women were identified from the family folder of the community health information system available at the local health post, with the support of Health Extension Workers working at the selected keble. |
| 06 | Include a citation to the Statistical Package for Social Science (SPSS) version 25 for data management | “(SPSS 25.0 Inc., Chicago, IL, USA)” |
| 07 | Translating the questionnaire into Amharic is excellent for local understanding, but it would be useful to mention if any back-translation process was conducted to ensure accuracy. | Yes!  The questionnaire was subsequently translated back into English to ensure its accuracy and consistency. |
| 08 | It would be helpful to describe how the pre-test sample was selected and whether it differed from the actual study population (e.g., in terms of socio-demographic characteristics). | We used 5% of our sample size (25 married women) to perform the pre-test in the Ambasel District, which is similar in socio-demographic characteristics to the study population. |
| 09 | Furthermore, it would be valuable to report the Cronbach's alpha result to indicate the tool's reliability. | The Cronbach’s alpha was calculated and found to be 0.799, confirming the reliability of the instruments used. |
| 10 | The study does not mention any checks for multicollinearity between independent variables, which could affect the validity of the regression results. | Multicollinearity among the independent variables was also assessed using the variance inflation factor (VIF), which was found to be less than 4.0. |
| 11 | While the study uses the Hosmer-Lemeshow test to check model fit, it does not mention the goodness-of-fit results. | The assumptions of logistic regression were checked using the Hosmer–Lemeshow goodness-of-fit test (Chi-square: 8.799, P-value: 0.36). |
| 12 | Describe the specific steps taken to protect data confidentiality, such as how data were stored and whether any identifiers were removed. | Respondents’ identifiers, such as names, were not requested or recorded during the interviews to protect data confidentiality. |

**Reviewer #3 Comments**

| **S.No** | **Comments** | **Response** |
| --- | --- | --- |
| 01 | Authors should consider placing findings from this study in context of findings not only from Ethiopia but also other countries in sub-Saharan Africa and countries like Ethiopia, globally. | Thank you for your concern and great suggestions.  We made comparisons with similar studies conducted in Sub-Saharan Africa countries. |
| 02 | Authors need to provide strong justification for why this study is necessary and the particular gaps that this study is filling. | Although numerous studies have explored the factors limiting women's autonomy in contraceptive use, women continue to face challenges in attaining full autonomy, as highlighted in a United Nations report. This study seeks to uncover additional factors contributing to the lack of autonomy among married women.  We considered women's freedom of movement and financial control when assessing married women's autonomy in the use of modern contraceptives. We also included non-users in our study, addressing gaps that most previous studies did not fully covered. |
| 03 | Provide references right after the sentences in lines 49, 51, and 54. | Thank for your suggestions.  We provided the references right after the sentences. |
| 04 | Line 94, “according to the United Nations..” | Thank you! We made the corrections. |
| 05 | Line 98, provide citations for these Ethiopian studies | Thank you! We provided the right citations. |
| 06 | Authors mentioned that “most Ethiopian studies..”. Highlight the Ethiopian studies that have looked at the variables: women’s freedom of movement and financial control | Thank you! We provided the right citations for those studies. |
| 07 | Household decision-making power, freedom of movement, and control over finance: were the questions pre-determined from the literature or were they created by researchers? | Thank you for your concern. The questions used to assess decision-making power, freedom of movement, and financial control variables were pre-determined in the EDHS 2016. |
| 08 | For the construct on “knowledge of modern contraceptive methods”: specify is this was binary (i.e., yes or no) or continuous and if so, what was the scale. | Yes! It was binary (yes or no). |
| 09 | Line 200: women who listened to the radio | Thank you! We made corrections based on your suggestions. *'Women who listened to the radio*' is grammatically correct and sounds more natural than *'women who heard radio*.' |
| 10 | For media exposure: did you consider social media? If not, consider this as a limitation in your discussion section. | Thank you. Since we did not consider social media in our assessment of media exposure, we have included this as a limitation, as per your suggestion. |
| 11 | Are there other studies that can be cited to show that unmarried women have more contraceptive use autonomy? | Yes! We have cited another study that supports this finding. |
| 12 | Why are you seeing higher numbers than reported by the EDHS for categories of women using contraceptives on the basis of their husbands’ decisions alone and those that stopped using because of their husbands’ decision alone? Provide more critical insights. | That was due to the fact that the national study in Ethiopia examined multiple regions, as well as differences in culture, educational attainment, media exposure, and wealth index across the various settings. Not only this, but also 67% of respondents in our study had been in their marital unions for less than 10 years, compared to 53% in the 2016 EDHS. As the duration of marital unions increases, women's decision-making autonomy regarding contraceptive use improves. |
| 13 | Provide references for line 343 | Thank you! We have provided the references. |
| 14 | Provide references for line 353-358 | Thank you! We have provided the references. |
| 15 | Provide references for lines 361- 364 “this might be because when a couple lives together for a longer period…” | Thank you! We have provided the references. |
| 16 | What are the implications of this study for global public health, policy making, community health? | Thank you for your great suggestion.  We have included the implications of the study in the discussion section. |
| 17 | Why is it important for women to use modern contraceptives over more indigenous ones? Western, modern versus traditional. What is the rationale here? | This is because, while traditional contraceptive methods play a role in family planning, their limitations in effectiveness and reliability warrant a focus on modern contraceptive methods |
| 18 | When you say women’s autonomy was low. What is the baseline for measurement here? | We concluded that women's autonomy was low based on the desired women's rights, but it was relatively higher compared to a recent national study in Ethiopia using the EDHS. |
| 19 | Future research may also consider how attitudes towards these contraceptives influence utilization and autonomy. | Thank you for your recommendations.  We have added, ‘’*Future research may also consider how attitudes towards these contraceptives influence utilization and autonomy*” in the conclusion section as a recommendation. |

**||Thank you||**
