# Supplementary material for: Married women’s autonomy in modern contraceptive utilization in Kutaber district, Northeast Ethiopia
Source: PLOS Glob Public Health. 2025 May 23;5(5):e0004671. doi: 10.1371/journal.pgph.0004671 (PMC12101775; doi:10.1371/journal.pgph.0004671)
Supplement: Attachment — Submitted filename: Response_to_Reviewers_auresp_2.docx [file pgph.0004671.s004.docx]

Date: March 19, 2025

**Rebuttal letter**

PGPH-D-24-02291

Married women’s autonomy in modern contraceptive utilization in Kutaber district, Northeast Ethiopia

PLOS Global Public Health

Dear Editor,

We would like to express our gratitude for your thorough evaluation of the manuscript during the review process. Both your comments and the reviewers’ have been carefully considered and incorporated, and all necessary amendments have been made. Below, we provide a point-by-point response to each comment. The specific modifications are highlighted via track changes in the attached files. As a result, this revised version of the manuscript has been significantly improved, and we anticipate it will now be suitable for publication.

We look forward to your response to ensure the manuscript progresses smoothly through the next stages.

Best regards,

Getachew Amanu

**Response to Editor’s Comment**

| **S.No** | **Comments/ Recommendations** | **Response** |
| --- | --- | --- |
| 01 | The autonomy score for current users is calculated differently to the scores for ever and non users.  For ever/non-users, a score of 1 means that decisions are made independently by the women or jointly with their husbands, whereas 0 means the decisions are made by someone else.  But for current users, you have split this first category (independent or joint) into two (1=joint, 2=independent).  The issue here is that there may not be equivalence between current users categorised as "autonomous" and past/non users.  It would make more sense to define what set of scores indicates autonomy (e.g., a score of at least one on all three questions). | Thank you!  We have accepted the issue and made revisions to the scoring and coding when measuring current contraceptive users to ensure equivalence between past users and non-users.  *A score of 1 was given to women who made decisions independently or jointly with their husband or someone else in the household. However, if the decision was made solely by the husband or another person in the household, the score was 0.*  *All participants who scored 1 on all three questions were categorized as “autonomous” and coded as 1, while those participants who scored 0 for any of the three questions were categorized as “not autonomous” and coded as 0.* |
| 02 | The 2016 EDHS defines participation in major household decisions as: "Women are considered to participate in household decisions if they make decisions alone or jointly with their husband in all three of the following areas: (1) the woman’s own health care, (2) major household purchases, and (3) visits to the woman’s family or relatives." | Great!  As per the comment, we measured women’s household decision-making power using three questions constructed based on items from the EDHS 2016. |
| 03 | The coding of household decision making power has the same issue.  What was the mean, and is it the case the categorisation accurately divides the participants into groups who do or do not have decision-making power? | *All participants who answered that they made decisions independently or jointly for all three items were categorized as “having household decision-making power” and coded as 1.*  *The participants who reported that another person made decisions for any of the three items were categorized as “not having decision-making power” and coded as 0.* |
| 04 | The same concern applies to the freedom of movement variable. | *"Participants who answered 'yes' to all three questions were categorized as 'having freedom of movement' and coded as 1. Those who answered 'no' to any of the three items were categorized as 'not having freedom of movement' and coded as 0.* |
| 05 | The same concern applies to control over finance variable. | *All participants who scored one on all four questions were considered to “have control over finance” and coded as 1. If not, they were considered to “have no control over finance” and coded as 0.* |
| 06 | It is therefore essential that you revise your coding and analyses so that the categories for each of the variables more accurately reflects the meaning of that category. | Thank you for your suggestions!  Following your constructive suggestion, after we made revisions to the scoring and coding for current contraceptive users, household decision-making power, freedom of movement, and control over finance variables, we conducted analysis again.  Therefore, the categories for each variable now more accurately reflect the meaning of that category. |

**Response to Reviewer III**

| **S.no** | **Comments/ Recommendations** | **Response** |
| --- | --- | --- |
| 01 | The study mentions discrepancies between its findings and Ethiopian Demographic and Health Survey (EDHS) 2016 data but does not fully explore potential explanations for these differences. A deeper comparison with more recent national surveys (e.g., the 2019 Mini DHS) could strengthen the discussion. | Thank you for your suggestion!  We cannot compare this study’s findings with the 2019 Mini EDHS because the latter does not include data on women’s decision-making power regarding family planning. |

**Thank you!**
